# West Nile Virus Transmission in Winter: The 2013 Great Salt Lake Bald Eagle and Eared Grebes Mortality Event

**DOI:** 10.1371/currents.outbreaks.b0f031fc8db2a827d9da0f30f0766871

**Published:** 2014-04-18

**Authors:** Hon S. Ip, Arnaud J. Van Wettere, Leslie McFarlane, Valerie Shearn-Bochsler, Sammie Lee Dickson, JoDee Baker, Gary Hatch, Kimberly Cavender, Renee Long, Barbara Bodenstein

**Affiliations:** USGS National Wildlife Health Center, Madison, Wisconsin, USA; Department of Animal, Dairy & Veterinary Sciences, Utah State University, Logan, Utah, USA; Utah Division of Wildlife Resources, Salt Lake City, Utah, USA; USGS National Wildlife Health Center, Madison, Wisconsin, USA; Salt Lake City Mosquito Abatement District, Salt Lake City, Utah, USA; Utah Department of Health, Salt Lake City, Utah, USA; Davis County Mosquito Abatement District, Kaysville, Utah, USA; Utah Division of Wildlife Resources, Salt Lake City, Utah, USA; USGS National Wildlife Health Center, Madison, Wisconsin, USA; USGS National Wildlife Health Center, Madison, Wisconsin, USA

**Keywords:** bald eagle, disease outbreak, eared grebe, Utah, West Nile virus

## Abstract

West Nile Virus (WNV) infection has been reported in over 300 species of birds and mammals. Raptors such as eagles, hawks and falcons are remarkably susceptible, but reports of WNV infection in Bald Eagles (Haliaeetus leucocephalus) are rare and reports of WNV infection in grebes (Podicipediformes) even rarer. We report an unusually large wild bird mortality event involving between 15,000-20,000 Eared Grebes (Podiceps nigricollis) and over 40 Bald Eagles around the Great Salt Lake, Utah, in November-December 2013. Mortality in grebes was first reported in early November during a period when the area was unseasonably warm and the grebes were beginning to gather and stage prior to migration. Ten out of ten Eared Grebes collected during this period were WNV RT-PCR and/or isolation positive. This is the first report of WNV infection in Eared Grebes and the associated mortality event is matched in scale only by the combined outbreaks in American White Pelican (Pelecanus erythrorhynchos) colonies in the north central states in 2002-2003. We cannot be sure that all of the grebes were infected by mosquito transmission; some may have become infected through contact with WNV shed orally or cloacally from other infected grebes. Beginning in early December, Bald Eagles in the Great Salt Lake area were observed to display neurological signs such as body tremors, limb paralysis and lethargy. At least 43 Bald Eagles had died by the end of the month. Nine of nine Bald Eagles examined were infected with WNV. To the best of our knowledge, this is the largest single raptor mortality event since WNV became endemic in the USA. Because the majority of the eagles affected were found after onset of below-freezing temperatures, we suggest at least some of the Bald Eagles were infected with WNV via consumption of infected Eared Grebes or horizontal transmission at roost sites.

## Introduction

West Nile Virus (WNV) was originally isolated from the blood of a febrile human patient in the West Nile district in Uganda. WNV is divided into four lineages (I-IV) 1. Lineage I viruses have been reported in Africa, the Middle East, the Mediterranean Basin, China and India; it is further divided into three clades (a-c), a clade 1a strain that is most closely related to 1998 isolates from Israel was introduced into the US in 1999. Sporadic introductions of Lineage I and II WNV continue to occur in the Mediterranean basin. Following its introduction into the New York area in 1999, WNV has become endemic and appears to have become more pathogenic to birds than its earlier relatives 2. In the New World, WNV shows a wide host range as it has been reported in over three hundred species of birds, in addition to a number of mammals, amphibians, and reptiles. In spite of this, WNV infection has been reported in only two species in the order *Podicipediformes *(grebes) 3 ^,^ 4. At least 36 species of raptors (orders *Falconiformes *and *Strigiformes*) are susceptible to infection with WNV 5. Species such as Red-tailed Hawks (*Buteo jamaicensis*) and Great Horned Owl (*Bubo virginianus*) develop systemic infections within five days post exposure, shed virus orally and cloacally and develop viremia capable of infecting mosquitoes (>10^5^pfu/ml serum). WNV infection can be a significant cause of raptor mortality; 46/132 (34.8%) of the WNV-infected raptors admitted to the Rocky Mountain Raptor Program between 2002-2005 had died or had to be euthanized 6. Of the raptors submitted to the National Wildlife Health Center between August-October 2002 for cause of death determination, 71% (40/56) were infected with WNV 7. Red-tailed Hawk is one of the species of raptor most often infected with WNV in Virginia and in 2003 alone, at least 90 died from WNV 8 ^,^ 9.

WNV is a member of the Flaviviruses, which are arboviruses that are usually transmitted by mosquitoes and ticks. However, WNV’s unusually broad number of transmission mechanisms includes blood transfusion, organ transplant, breast milk, transplancental transmission, percutaneous (broken skin or mucosa), and respiratory exposure in humans 10 ^,^ 11 ^,^ 12. In birds, besides inducing a viremic phase in susceptible species that is sufficient to infect additional mosquitoes, WNV can also be shed orally and cloacally, giving rise to the possibility of transmission mechanisms beyond conventional vector-borne transmission 13. Oral and respiratory infection routes may be a common feature of many arboviruses, and transmission by ingestion of WNV-infected carcasses has been demonstrated experimentally 13 ^,^ 14 ^,^ 15 ^,^ 16. Birds can be persistently infected and harbor WNV for several weeks to months following exposure and may be a source of WNV after mosquito activity ceases 17 ^,^ 18.

Half of the entire North American population of Eared Grebes gather at the Great Salt Lake from August to December 19. The grebes undergo a profound physiological change; their pectoral and cardiac muscles atrophy and they become flightless; leg muscles and digestive organs develop to enable the diving and digestion of an estimated 26,500-29,600 brine shrimp (*Artemia franciscana*) per day to accumulate energy necessary for migration to the southern US and Mexico 20 ^,^ 21. The birds double in weight, and before departure, a reverse reorganization occurs: the digestive organs shrink, leg muscle mass decreases, and flight and cardiac muscles redevelop. The reorganization is triggered when the population of brine shrimp declines to unexploitable levels 21. This pattern of fall staging makes the autumn migration of the Eared Grebes the latest of any bird species in North America 22. 25-30% of the Bald Eagle population that winters in the lower 48 states west of the Rocky Mountains are found in Utah. The Great Salt Lake area hosts one of the largest populations of Bald Eagles in the state, with an estimated 750-1200 eagles arriving in November through mid-February 23.

We describe an unusual late season (Nov-Dec 2013) transmission of WNV as the likely cause of a large-scale wild bird mortality event at the Great Salt Lake, UT, involving two species, the Bald Eagle and Eared Grebe. The die-off may be the largest documented wild bird mortality event due to WNV in the US and is the largest WNV-associated raptor mortality event since WNV became endemic in the US. (We defined an event as an outbreak of a specific duration and circumscribed in area.) Here we describe the epidemiology of the this outbreak as of 2013 and the results of investigations to date, and we discuss factors that may have contributed to the occurrence of the outbreak.

## Materials and Methods

Specimen Collection. Birds found dead or euthanized between Nov 1 and Dec 31, 2013, in the Great Salt Lake area (approx. Latitude 41.16°, Longitude -112.58°) were collected by the Utah Division of Wildlife Resources and submitted to the Utah Veterinary Diagnostic Laboratory (UVDL), Logan, UT, and to the USGS National Wildlife Health Center (NWHC), Madison, WI, for complete diagnostic analysis. Post-mortem (necropsy) examination was performed and a complete set of tissues was fixed in 10% neutral buffered formalin, processed, and embedded in paraffin for routine histopathological analysis.

Laboratory Testing and Virus identification. Brain, liver and heart were submitted for bacterial culture by routine laboratory procedures using sheep blood agar, MacConkey agar, and Columbia CNA agar. Testing for*Chlamydophila/Chlamydia*spp. by PCR was performed in one bird by the Wisconsin Veterinary Diagnostic Laboratory. To evaluate for the possibility of organophosphate and carbamate insecticides, brain acetylcholinesterase activities were measured 24. Samples of liver were collected during necropsy, saved frozen, and analyzed for lead concentration using atomic absorption spectroscopy (UVDL and NWHC). Tracheal and cloacal swabs from all eagles were tested for Avian Influenza and Avian Paramyxovirus-1 (Newcastle Disease Virus) using RT-PCR tests 25 ^,^ 26. A 10% homogenate of suspect tissues was filtered through a 0.22 mm syringe filter and the RNA extracted using commercial kits. The RNA was tested for the presence of WNV as described by Lanciotti et al. 27. RT-PCR positive samples were inoculated into Vero tissue culture cells as described by Docherty et al. 28. The identity of suspect WNV isolates was confirmed by the same RT-PCR test.

## Results

Beginning in November 2013, Eared Grebes were found dead on the lake with others exhibiting clinical signs that included inability to hold head erect, lethargy and inability to dive. Mortality was first noted on Nov 8, and as of Dec 31, 2013, approximately 15,000-20,000 grebes had died. A total of ten Eared Grebes collected in 2013 have been examined at NWHC. Five were collected on Nov 21 in Box Elder County and five on Dec 20 from Davis County (Fig. 1, Table 1). Based on gross presentation, avian cholera was suspected in four birds, cardiomyopathy in two birds, ulcers in two birds, and enteritis, and trauma (from bite/talon wounds) in one bird each. Histological examination on two of these birds revealed encephalitis and myocarditis. All birds examined tested negative for lead poisoning. No significant pathogenic bacteria or fungi were cultured from any of the grebes examined. Avian influenza and Avian Paramyxovirus 1 (Newcastle Disease Virus) RT-PCR tests were negative on all tracheal and cloacal swabs. WNV was detected in all ten grebes (Table 1). WNV was isolated from seven individuals, including two out of four livers, five out of five kidney/spleen pools, six out of six brains, and the heart and feather pulp from one grebe. WNV shedding as detected by RT-PCR was found in seven out of ten cloacal swabs and in three of ten tracheal swabs.

**Utah counties with reported mortality of birds at Great Salt Lake in December 2013.**
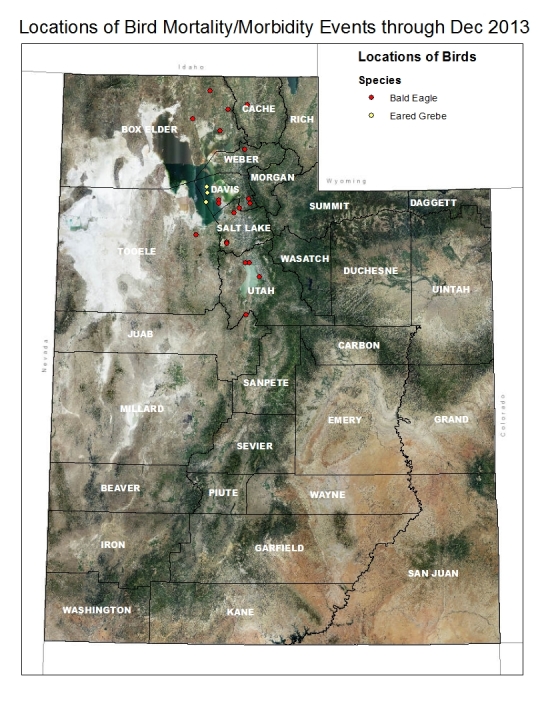
Location of sick or dead birds during the Great Salt Lake WNV mortality event are color coded in red for bald eagles and yellow for eared grebes. The counties in Utah are labeled in white.

**Cumulative mortality of bald eagles over time during the Great Salt Lake WNV mortality event.**
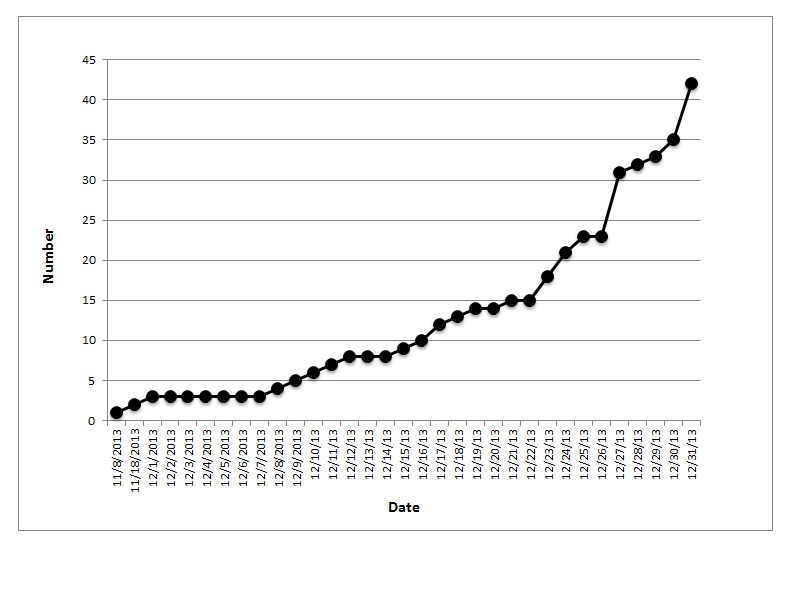
The number of bald eagle deaths in the Great Salt Lake area during December 2013 are plotted against dates.

Bald Eagles found dead or presenting with head tremors, paralysis of the wings and legs and lethargy were observed in the Great Salt Lake area beginning in December 2013. The index case, a Bald Eagle from Weber County, was admitted to a wildlife rehabilitation facility on Dec 1 and died on Dec 9. A total of 43 Bald Eagles from six counties (Box Elder, Davis, Salt Lake, Tooele, Utah and Weber) died or were euthanized between Dec 1 and Dec 31 (Fig 2).

As of Jan 5, 2014, a total of eight Bald Eagles have been submitted to the NWHC and one to the UVDL for postmortem examination (Table 1). All birds were in fair to excellent postmortem condition and nutritional status, with the exception of one Bald Eagle that was in poor nutritional status. Initial differential diagnoses based on gross presentation in the Bald Eagles were lead poisoning or WNV. Histological examination of six birds revealed meningoencephalitis and myocarditis in all birds. No bacteria including *Pasteurella multocida*, *Erysiplothrix *or *Salmonella *sp. were obtained from the Bald Eagles’ liver. One Bald Eagle had *Aspergillus *isolated from lung tissue. The spleen from one Bald Eagle tested negative for *Chlamydophilia/Chlamydia *by PCR. Eight Bald Eagles were negative for exposure to lead, and one Bald Eagle had a blood lead level of 0.28 ppm. All Bald Eagles were negative for exposure to organophosphate insecticides.

Nine out of nine (100%) brains and four of four (100%) kidney/spleen pools tested positive for WNV by rRT-PCR. A WNV isolate has been isolated from the brains of eight eagles and from the kidney/spleen pool from three of four eagles (Table 1).

**Figure:**
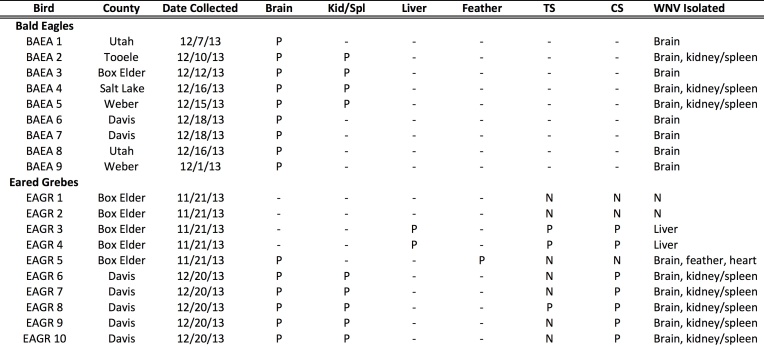
**Table 1.** Bird. BAEA, Bald Eagle, EAGR, Eared Grebes. County, county of collection. Date Collected, date when bird was initially collected. WNV RT-PCR results from Brain, Kid/Spl (kidney/spleen pool), Liver, Feather pulp, TS (tracheal swab), CS (cloacal swab) are listed as P, positive (Ct < 40), N (Ct = 0), or – (not done). WNV Isolated, tissues from which a WNV has been isolated is listed, N is no virus isolated.

## Discussion

**WNV in Eared Grebes**

At the Great Salt Lake, we have detected WNV in two groups of Eared Grebes collected a month apart and from two different locations, suggesting that exposure to WNV might have been widespread in this species at this location in 2013. To the best of the authors’ knowledge, the Great Salt Lake mortality event is the first time that WNV infection in Eared Grebes has been reported. It is unclear at the present time if the entire 15,000-20,000-grebe mortality (out of an estimated population of 2 million grebes at the time) is due to WNV. On a broader geographic scale and over a longer period of time, more than 9,000 American White Pelicans died over seven states in 2002 and as much as 45% of the US crow population may have died by 2005 following the introduction of WNV in 1999 9 ^,^ 24 ^,^ 29 ^,^ 30, but if all of the dead Great Salt Lake grebes did die of WNV in 2013, as seems likely from our consistent laboratory findings, this might be the single largest WNV-associated wild bird mortality event, occurring as it did over the course of two months and being restricted to the Great Salt Lake area. Grebe die-offs in such numbers are more typical of avian cholera (*Pasteurella multocida*), which is known to occur in this area. However, avian cholera was not involved in the deaths of any of the grebes examined in 2013. Inoculation trials will be necessary to determine the relative susceptibility of grebes to WNV infection. Historically, WNV infection in waterfowl (*Anseriformes*) is uncommon and in waterbirds of the family *Podicipediformes*, it has been reported only in a Pied-billed Grebe (*Podilymbus podiceps*) from a small-scale mortality event at Agassiz National Wildlife Refuge, MN, in 2002 and in a Clark’s Grebe (*Aechmophorus clarkii*) collected as part of Colorado’s wild bird WNV surveillance program 31 ^,^ 32.

**WNV in Bald Eagles**

Bald Eagles began to migrate into the Great Salt Lake region in November, and neurological signs were observed soon after their arrival. We have found WNV in the brain and other tissues in each of the Bald Eagles tested. Early in the outbreak, there were concerns that the mortality in this area was due to lead poisoning or avian cholera or avian vacuolar myelinopathy, based on past history in this species. The consistent identification of WNV in the eagles and the absence of other infectious diseases or indications of poisoning led us to conclude that WNV, even though the timing of the outbreak was unusual, was the causative agent in the death of the Bald Eagles that died in the Great Salt Lake area in Dec 2013. Little is known about the course of WNV infection in Bald Eagles, but the presentation of encephalitis and myocarditis is typical of WNV infection in susceptible species 33. While WNV infection has been reported in over 36 species of raptors, records of WNV in Bald Eagles are rare 5 ^,^ 7 ^,^ 34. Excluding the current outbreak in Utah, a total of 379 Bald Eagles collected nationwide have been tested to date at NWHC since 1999, and WNV isolated from 11 (2.9%) (NWHC, unpublished). In contrast, a higher prevalence of WNV is reported in other raptor species. For example, in nearby Colorado, 36.4% (64/176) of raptors [American Kestrel (F*alco sparverius*), Red-tailed Hawks (*Buteo jamaicensis*) and Great Horned Owl (*Bubo virginianus*)] in 2003 were infected with WNV 31. Whether the low number of WNV-positive Bald Eagles reported prior to the present outbreak is due to innate resistance, lack of exposure to infected vectors, lack of reporting, or other factors remains to be determined.

**Timing of the WNV outbreak**

Eared Grebes are almost entirely aquatic. After the post-breeding season, grebes begin to migrate to the Great Salt Lake in August from across eastern and central North America. Eared Grebes have distinct daily and seasonal movement patterns, moving in shore before sunrise to feed and after a few hours withdrawing up to 1-2 km off shore to preen and rest. The flock returns to the shallows to feed in the late afternoon and moves off to roost at dusk 22. As much as 74% of the daylight hours are spent in diving bouts in search for forage 20.

The most common mosquito species in the Great Salt Lake area (and in the rest of the state) is *Culex tarsalis*, a proven vector of WNV. *Culex erythrothorax *is common in the marshes surrounding Great Salt Lake and is a competent vector of WNV 35 *.* *Cx. erythrothorax *population peaks in autumn, and the adults of this species do not enter diapause and continue to bite into late autumn when *Cx. tarsalis *has stopped. Grebe distribution on the lake is not uniform, and most tend to concentrate in the South Arm, where it is less saline and has higher shrimp concentration. This location brings the birds in close proximity to wetlands such as those in Davis County and well within the 12.6 km maximal flight distance recorded by *Culex *mosquitoes 36. The frequency of contact between grebes and mosquitoes on Great Salt Lake has not been studied, but the diurnal movement of the grebes is likely to bring them in and out of several mosquito vectors’ ranges.

WNV activity in the whole state, and in the Great Salt Lake region in particular, was higher in 2013 than in 2012, but the activities in both years were significantly lower than the levels of activity seen in 2006 37 ^,^ 38. WNV was found in 2013 in all the counties where Bald Eagle and Eared Grebes had tested positive for WNV during this event. Thus, we cannot exclude some level of autochthonous WNV exposure to grebes in Great Salt Lake between August and mid-October 39, but it seems unlikely that mosquito transmission of WNV was sufficiently intense prior to December 2013 to directly cause the death of so many grebes without other indications in the State of Utah’s WNV monitoring program.

**Possible Transmission Mechanisms**

Unusual behavior and deaths in grebes was first reported at the Great Salt Lake in early November 2013, and the first Bald Eagle was admitted to the rehabilitation center on December 1. Although mosquito activity is generally absent at this time of year, the weather around the Great Basin was unusually warm in the autumn of 2013, with daily maximum temperatures falling below 10 °C consistently only after Dec 3 (Fig. 3). As 10 °C marks the approximate temperature at which most mosquitoes, but not *Cx. erythrothorax*, enter diapause, active vector-borne WNV transmission might have occurred in October or continued to occur in November. This might be one mechanism by which the Eared Grebes became infected. Moreover, WNV can overwinter in some mosquito species, and while the temperature is below that necessary for extrinsic replication of the virus in newly infected mosquitoes, those infected earlier in 2013 may remain capable of infecting grebes and eagles in November 40 ^,^ 41.

**Daily maximum and minimum air temperature record at the NOAA Great Salt Lake meteorological station.**
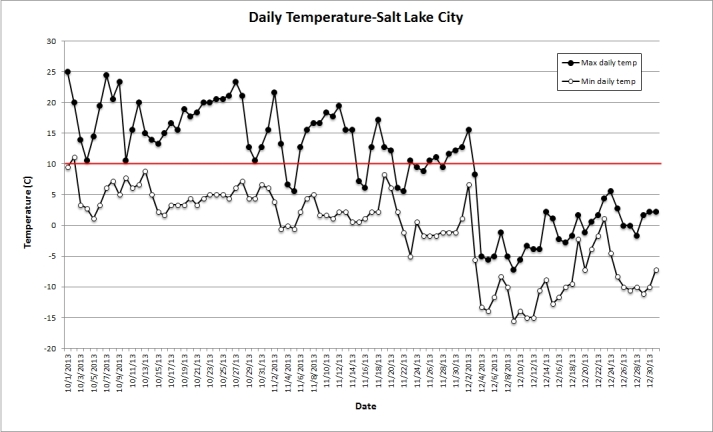
The maximum (filled circles) and minimum (open circles) daily temperatures at the Salt Lake City Meteorological Station from October 1-December 31, 2013 are plotted against dates. The average temperature (10°C) for resumption of mosquito activity is marked in red.

The congregation of an increasing number of grebes in late fall in small areas of high brine shrimp concentration may have allowed for further horizontal transmission to grebes via shedding of the WNV cloacally 42. Contact or fecal-oral transmission has been suspected when uninfected crows co-housed with experimentally infected crows died of WNV 43. This mechanism is suspected to have occurred naturally in the deaths of crows from WNV in New York between Feb 10-Mar 29, 2004 44. Bird-to-bird transmission has been identified as a major factor in the spread of WNV when mosquito-to-bird ratio is low 45. Wintering Bald Eagles share roosts in communal trees, which brings them into close proximity. Transmission by ectoparasites and/or from fomites, such as roosts contaminated with infected feces, are remote, but possible, transmission mechanisms. Bald Eagles also practice “kleptoparasitism” or interspecies piracy, where birds actively steal food from each other. One study found that 5.7% of the daylight hours are spent stealing 46. A carcass contaminated by the feeding activity of an infected eagle might infect a second eagle if stolen 47. In December 2013, following onset of colder temperatures, mosquito-mediated transmission likely ceased, and local hunters report that the ice-covered lakes and ponds in the adjacent area precluded Bald Eagles from fishing for one of their major food sources, carp. The eagles were forced to forage further afield and could have been exposed to WNV by scavenging on infected grebes’ carcasses. Little is known as to the survival of WNV in a carcass under the conditions of cooler weather, but the lower temperature is likely to enhance survival. Scavenging of WNV-infected carcasses has been proposed as a potential mechanism of WNV transmission 16, and the present outbreak, especially in Bald Eagle cases in the second half of December, might have resulted from scavenging of infected grebe carcasses. A single Red-tailed Hawk found dead in February 2002 in Westchester, NY, and from whose brain WNV was isolated, was thought to have been infected by eating an infected prey 47.

We suggest that the “perfect storm” of the combination of late season mosquito activity from the warm temperature, congregation of large concentrations of grebes, horizontal transmission from oral and/or cloacal shedding of WNV from infected grebes and eagles, and transmission by ingestion of infected carcasses together contributed to the unusual WNV mortality in the Eared Grebes and Bald Eagles around the Great Salt Lake in 2013. This brief report provides some preliminary findings. Additional research such as full histopathological and immunohistochemical description, virus sequencing to determine viral origin and possible genetic mutations that might be associated with increased virulence, and experimental infection trials to demonstrate Koch’s postulate and other studies remain to be done.

As of the beginning of 2014, the mortality event in both species was diminishing, but monitoring will continue at Great Salt Lake and at the migratory destinations of grebes for continued/additional outbreaks. With the WNV-related deaths of Eared Grebes and Bald Eagles in the winter months, concern is raised that this situation is a significant departure from what has been observed in the recent past in Utah. If *Cx. erythrothorax *is involved, WNV may overwinter in this species as the virus was detected in *C. erythrothorax *larvae collected on Oct 28, 2004 in Moab, UT 35. The combination of novel susceptible species, late season transmission, and virus overwintering leads to further concerns that WNV may now play a larger role in human, horse and bird health in Utah in the future.

## Competing Interests

The authors have declared that no competing interests exist.
